# Environmental Intolerance, Symptoms and Disability Among Fertile-Aged Women

**DOI:** 10.3390/ijerph15020293

**Published:** 2018-02-08

**Authors:** Aki Vuokko, Kirsi Karvala, Jussi Lampi, Leea Keski-Nisula, Markku Pasanen, Raimo Voutilainen, Juha Pekkanen, Markku Sainio

**Affiliations:** 1 Finnish Institute of Occupational Health, P.O. Box 40, 00032 Helsinki, Finland; kirsi.karvala@ttl.fi (K.K.), markku.sainio@ttl.fi (M.S.); 2 Environmental Health, National Institute for Health and Welfare, P.O. Box 95, 70701 Kuopio, Finland; jussi.lampi@thl.fi (J.L.); juha.pekkanen@helsinki.fi (J.P.); 3 Social and Health Services, 70701 Kuopio, Finland; 4 Department of Obstetrics and Gynecology, Kuopio University Hospital, P.O. Box 100, 70029 KYS Kuopio, Finland; leea.keski-nisula@kuh.fi; 5 Institute of Clinical Medicine, School of Medicine, University of Eastern Finland, P.O Box 1627, 70211 Kuopio, Finland; raimo.voutilainen@kuh.fi; 6 School of Pharmacy, Faculty of Health Sciences, University of Eastern Finland, P.O. Box 1627, 70211 Kuopio, Finland; markku.pasanen@uef.fi; 7 Department of Pediatrics, Kuopio University Hospital, P.O. Box 100, 70029 KYS Kuopio, Finland; 8 Department of Public Health, University of Helsinki, P.O. Box 20, 00014 Helsinki, Finland

**Keywords:** idiopathic environmental intolerance, multiple chemical sensitivity, sick building syndrome, electromagnetic hypersensitivity

## Abstract

The purpose was to study the prevalence of environmental intolerance (EI) and its different manifestations, including behavioral changes and disability. Fertile-aged women (*n* = 680) of the Kuopio Birth Cohort Study were asked about annoyance to 12 environmental factors, symptoms and behavioral changes. We asked how much the intolerance had disrupted their work, household responsibilities or social life. We chose intolerance attributed to chemicals, indoor molds, and electromagnetic fields to represent typical intolerance entities. Of the respondents, 46% reported annoyance to chemicals, molds, or electromagnetic fields. Thirty-three percent reported symptoms relating to at least one of these three EIs, 18% reported symptoms that included central nervous system symptoms, and 15% reported behavioral changes. Indicating disability, 8.4% reported their experience relating to any of the three EIs as at least “somewhat difficult”, 2.2% “very difficult” or “extremely difficult”, and 0.9% “extremely difficult”. Of the latter 2.2%, all attributed their intolerance to indoor molds, and two thirds also to chemicals. As the number of difficulties increased, the number of organ systems, behavioral changes and overlaps of the three EIs also grew. EI is a heterogeneous phenomenon and its prevalence depends on its definition. The manifestations of EI form a continuum, ranging from annoyance to severe disability.

## 1. Introduction

Intolerance to environmental factors at very low exposure levels is a frequently encountered health issue. This intolerance varies from annoyance to disabling multiple organ symptoms causing lifestyle changes and functional impairments [1,2]. Environmental intolerance (EI) is an acquired condition, often attributed to various odorous substances (e.g., vehicle exhaust, perfumes, cigarette smoke, cleaning agents, freshly printed papers), indoor air environments (certain buildings), and electromagnetic fields (EMFs) (electrical devices) [1,2,3,4,5,6]. A common consequence of all types of EI is that individuals become sick from exposure below hazardous or toxic levels, although some individuals get symptoms while others do not, despite being exposed to the same environment [4,7,8]. These adverse health effects cannot be explained by known toxicological, allergological or physical mechanisms [9], and consistent evidence shows that nocebo mechanisms are involved [8].

A workshop organized by the World Health Organization (WHO) and two other United Nations (UN) agencies [3] stated that medically unexplained conditions attributed to diverse environmental factors should be labeled under the same term, idiopathic EI, due to their similar features. Such conditions include multiple chemical sensitivity (MCS), hypersensitivity to EMFs and sick building syndrome (SBS) [2,3,4,5]. SBS is characterized by non-specific building-related symptoms with often an unclear cause, but with a possible relation to the indoor environment [5,10,11,12]. SBS, among other EI, represents ill health that is more serious than that which could be reasonably expected to result from exposure.

EI is a heterogeneous condition with different degrees of severity that have been shown in clinical settings [13]. In population-based studies, the spectrum of EI is even wider and includes mild sensitivities such as annoyance to everyday odors, and is reflected by greatly varying prevalence rates [14]. Most prevalence data are from studies on EI attributed to chemicals with prevalence ranging from 0.5% to 52%, depending on the population and case definition [1,15,16,17,18,19,20,21]. The prevalence of EI attributed to EMFs varies between 1.5% and 21% [6,7,22]. However, studies on the prevalence of EI attributed to certain buildings are scarce. In a Swedish population-based study, 18% of the participants reported one or more symptoms compatible with SBS (mucosal, skin, or general symptoms) at work or in the home environment [23] and in another study this proportion was 21% [24]. In population-based samples, the prevalence of EI attributed to certain buildings was 7.1% in Finland and 4.8% in Sweden [14].

Prevalence data on EI with disability is fragmented and severity is described non-uniformly. The severity of EI has been described by the degree of annoyance [16], severity or strength of symptoms [15,19], number of symptom groups [24], lifestyle and behavioral changes [1,20,25], or whether the condition has been diagnosed by a physician (e.g., [6]). In the study of Berg et al. [1] on a sample based on the general population, 3.3% had made adjustments to their social lives or occupational conditions, 27% reported symptoms due to intolerance to inhaled chemicals, and 45% reported annoyance. The prevalence of self-reported physician-diagnosed EI varies from 0.5% to 6.3% [6,14,15,18,20]. A physician’s diagnosis represents a condition requiring medical attention and probably more severe EI.

Palmquist et al. [6] showed co-occurrence of EI attributed to chemicals, certain buildings, and EMFs, strengthening the hypothesis that different types of EI share similar mechanisms and are likely to represent the same phenomenon. A typical feature of all EIs is that they are reported significantly more often by women than men [2,8,23].

Previous data describe certain features of EI, but the different degrees of the various types of EI and their co-occurrence are still not understood.

The aim was to study the prevalence of self-reported EI and its different manifestations ranging from annoyance to a more severe condition in a female population, utilizing a maternity clinic cohort. We studied if symptoms, behavioral changes, and the co-occurrence of different types of EI associate with the severity of intolerance.

## 2. Methods

### 2.1. Study Design and Study Population

This cross-sectional questionnaire study was part of the ongoing Kuopio Birth Cohort (KuBiCo), which studies the effect of different risk factors on the health of the mother and the child. The participants are recruited from maternity clinics that serve all women who give birth at Kuopio University Hospital, which is the main maternity hospital in Eastern Finland and has about 2000–2500 deliveries annually. In Finland, in practice all pregnant women regardless of their socioeconomic status attend municipal maternity clinics that provide guidance in all matters related to pregnancy. Study participants were recruited via a web-based platform, which was used by more than two thirds of pregnant women.

In Finland, exposure to environmental tobacco smoke at public and work places is restricted by a smoking ban. In the study region, 1.6% of women report exposure at home and 2.4% at work. 

An electronic questionnaire on EI was offered in the first trimester to all Finnish-speaking pregnant women who participated in the KuBiCo Study during the period from July 2012 to February 2014. Altogether 680 women participated in the EI study. An exact participation rate cannot be given. Based on 2500 annual deliveries and taking into account the fact that the questionnaire was available to two thirds of the maternity clinic clients, approximately 27% of which were recruited for this study. 

The study was approved by the Ethics Committee of the Hospital District of Central Finland, Jyväskylä (dated 15.11.2011).

### 2.2. Intolerance Attributed to Environmental Factors

The questionnaire was designed to assess the increasing severity of intolerance attributed to certain environmental factors and associated symptoms, behavior changes, and disability (Table 1). The respondents were asked to apply their evaluations to the time prior to their pregnancy.

We asked the participants to rate their annoyance attributed to 12 different environmental factors on a scale from 0 (not at all) to 3 (very much). Only ratings of 2–3 (“rather much” or “very much”) were taken into account in the EI definitions (A–F, see below).

Using an additional question, we inquired how sensitive the respondents considered themselves, ranging from 1 (not at all) to 4 (very sensitive). Those who reported annoyance attributed to at least one of the 12 environmental factors, but considered themselves not at all sensitive (*n* = 50), were excluded from further questions on symptoms, behavioral changes and disability.

#### 2.2.1. Symptoms

The respondents were asked if they had intolerance-associated symptoms from the following symptom groups: neurological, cognitive, pulmonary, dermal, musculoskeletal, gastrointestinal, cardiac, and general symptoms, according to Black et al. [26] (Table 1). The response options for each item were “yes” or “no”. Neurological and cognitive symptoms were regarded as central nervous system (CNS) symptoms; the rest of the symptoms were divided into seven different organ systems.

#### 2.2.2. Behavioral Changes

Intolerance-associated behavioral changes were asked with the question “Have you made any behavioral changes to avoid the above symptoms?”, which was modified from Black et al. [26]. Response options for each item (Table 1) were “yes” or “no”. 

#### 2.2.3. Disability

To assess the severity of EI (how much the intolerance had disrupted work, household responsibilities or social life) we used a single item from the Patient Health Questionnaire (PHQ-9) [27] as a measure of disability, with a scale of “not difficult at all”, “somewhat difficult”, “very difficult”, and “extremely difficult” (Table 1).

### 2.3. Definitions of EI

We used the following EI definitions to identify different degrees of EI that represent increasing severity and strictness of the criteria:

(A) Feeling ill or annoyed (annoyance) by different environmental factors;
(B) Annoyance with symptoms;
(C) Annoyance with symptoms from multiple organ systems including the CNS (at least one CNS symptom and one non-CNS symptom);
(D) Annoyance with multiple organ symptoms including CNS symptoms (=definition C) and behavioral changes (at least one behavioral change, see Table 1);
(E) Annoyance with multiple organ symptoms including CNS symptoms, behavioral changes (=definition D) and disability; and;
(F) Annoyance with multiple organ symptoms including CNS symptoms, behavioral changes (=definition D) and severe disability.

“Somewhat difficult”, “very difficult” and “extremely difficult” responses to the disability question represented disability in EI definition E. In EI definition F, “very difficult” and “extremely difficult” were combined to represent severe disability. Definition E was based on the latest MCS criteria update [4].

### 2.4. EI Attributed to Chemicals, Indoor Molds, and EMFs

Three types of EI corresponding to MCS, non-specific building related symptoms (SBS), and EMF-hypersensitivity were studied as follows: EI attributed to chemicals was determined if the respondent reported intolerance (“rather much” or “very much” annoyance) to two or more of six chemical items (Table 1, items 1–6), according to Black et al. [26]. EI attributed to indoor molds was defined by reported intolerance to “indoor molds in moisture-damaged buildings”. We chose indoor molds to represent building-related intolerance because, of all indoor air-related factors, people consider molds the major health hazard in Finland. EI attributed to EMFs was defined by intolerance to EMFs (Table 1).

### 2.5. Statistical Analysis

Statistical analyses were performed using IBM-SPSS Version 24.0 for Windows (SPSS, Chicago, IL, USA) software. We used the Kruskal-Wallis test to compare the grade of disability with the number of organ systems, the number of behavioral changes, and co-occurrence of the three EIs, and to compare the three types of EI with the number of organ systems. We also used the Kruskal-Wallis test to compare the increasing severity of the continuum of EI (definitions A–F) with the co-occurrence of the three EIs. For the categorical variables, we used the χ^2^ test. The level of significance was set at *p* < 0.05. If an individual fulfilled the stricter criteria for EI, she was also included in the lower severity EI definitions, e.g., an individual fulfilling definition F (high intolerance) criteria belonged also to EI definitions A to E. In the analyses, an individual was included only once. Co-occurrence of EI attributed to chemicals, indoor molds, and EMFs are shown with Venn diagrams. We calculated the prevalence values for these three EIs (with or without co-occurrence) by proportions expressed as percentages of the sample. 

## 3. Results

Altogether 680 women, aged 16–45 (mean 29.9, standard deviation (SD) 4.8) participated in the study. The majority, 90.9% (*n* = 618) reported being non-smoking. Almost all, 94.9% (*n* = 645) reported being at least somewhat annoyed by at least one of the inquired 12 environmental factors (Table 2).

### 3.1. Annoyance Attributed to Environmental Factors

The prevalence of EI according to definition A was 67.2% (*n* = 457) (“rather much” or “very much” annoyance attributed to at least one of the 12 presented environmental factors) (Table 3). The prevalence of annoyance attributed to chemicals (two or more of the selected six chemicals), indoor molds, and EMFs were 29.1%, 32.6%, and 2.9%, respectively (EI definition A) (Table 3).

### 3.2. Annoyance and Symptoms Attributed to Environmental Factors

**Figure 1:**
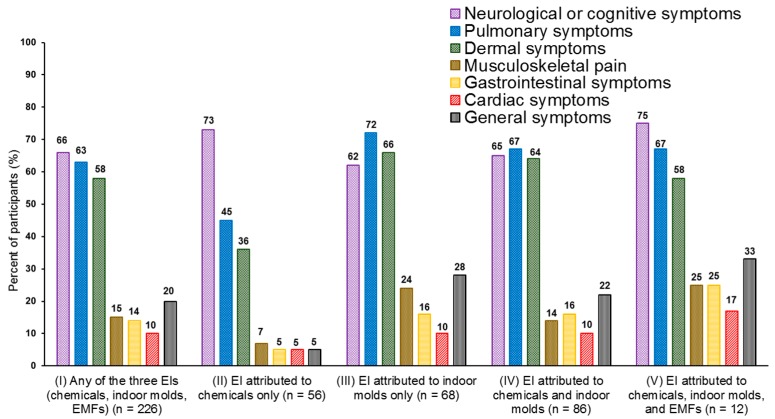
Proportion of self-reported symptoms in different organ systems among cases * with environmental intolerance (EI) attributed to chemicals **, indoor molds, electromagnetic fields (EMFs), and their combinations. Symptoms were assigned to seven organ systems. An individual may have symptoms from one or more organ system. Mean numbers (SD) of organ systems (1–7) were 2.5 (1.5) for (I), 1.8 (1.1) for (II), 2.8 (1.7) for (III), 2.6 (1.6) for (IV), and 3.0 (1.5) for (V). * Those intolerant (*n* = 226) who reported symptoms in one or more organ system (EI definition B); ** Two or more of the six chemicals in Table 1.

Annoyance with one or more symptoms (EI definition B) was attributed to chemicals by 22.8%, to indoor molds by 24.4%, to EMFs by 2.4%, and to any of these three EIs by 33.2% (*n* = 226) of the participants (Table 3). Of these 226 participants, 38.9% reported symptoms in three or more different organ systems. The symptoms occurred predominantly in the CNS, pulmonary tract, and dermal systems (Figure 1). Those with EI attributed to chemicals but not EMFs or molds (*n* = 56) had significantly fewer symptoms in the pulmonary tract (*p* = 0.001) or dermal system (*p* < 0.001), fewer general symptoms (*p* = 0.002), musculoskeletal symptoms (*p* = 0.047) or gastrointestinal symptoms (*p* = 0.036), and symptoms in a smaller number of organ systems (*p* < 0.001) than those with any EI (*n* = 170) (Figure 1). Musculoskeletal (*p* = 0.028) and general symptoms (*p* = 0.047) were significantly more common among those intolerant to only molds and not chemicals or EMFs (*n* = 68), than among those with any EI (*n* = 158).

The prevalence of annoyance with multiple organ symptoms, including CNS symptoms (EI definition C) is shown in Table 3. 

### 3.3. Behavioral Changes Due to EI

Of all the participants, 9.9% reported annoyance to chemicals and behavioral changes to avoid symptoms (EI definition D), 12.2% to indoor molds, and 1.3% to EMFs (Table 3). The respondents who had at least one of the three EIs according to EI definition D (*n* = 102) reported having made the following behavioral changes: behavioral or lifestyle change to minimize exposure (*n* = 65), changed interior decorations or furnishings at home (*n* = 29), moved to another apartment (*n* = 24), changed workplace, resigned from workplace or occupation (*n* = 19), taken vitamins, supplements, or changed diet (*n* = 47), eliminated the cause using antifungal agents or chemicals (*n* = 16), and used protective equipment (*n* = 55). Nine respondents (1.3%) reported both a move to another apartment and a change of workplace.

### 3.4. Disability Due to EI

Of all the respondents, 5.7%, 7.6%, and 0.7% reported at least a “somewhat difficult” disability (EI definition E) due to EI attributed to chemicals, indoor molds, and EMFs, respectively (Table 3). At least somewhat disability (EI definition E) attributed to any of the three EIs was reported by 8.4%, and severe disability (EI definition F) was reported by 2.2% (*n* = 15) (Table 3). All 15 participants with severe disability attributed the intolerance to indoor molds and 10 (1.5%) attributed it to molds and chemicals (Table 3 and Table 4). Of these 15 participants, 12 reported having had to change apartment or job to avoid symptoms due to intolerance: had moved to another apartment (*n* = 9), changed workplace, resigned from their workplace or occupation (*n* = 7), and four participants reported having done both. Of the 15 participants, all reported CNS symptoms, 80% (*n* = 12) dermal symptoms, and 73% (*n* = 11) pulmonary symptoms. The mean number of organ systems presenting symptoms was 4.4 (SD 2.0; range 2–7).

Among the respondents having symptoms due to chemicals, molds, or EMFs (EI definition B), an association was found between more severe disability and pulmonary tract symptoms (*p* = 0.011), and nearly significantly for CNS symptoms (*p* = 0.054). More severe disability was associated with a higher number of organ systems being involved (EI definition B, *p* < 0.001), and with a higher number of behavioral changes (EI definition B, *p* < 0.001; EI definition C, *p* = 0.001).

Of all the 680 respondents, six (0.9%) reported very severe (“extremely difficult”) disability (Table 4). All these six had CNS and pulmonary tract symptoms, and five had dermal system symptoms. The mean number of organ systems involved was 4.8 (SD 2.1; range 2–7).

### 3.5. Co-Occurrence of EIs

**Figure 2:**
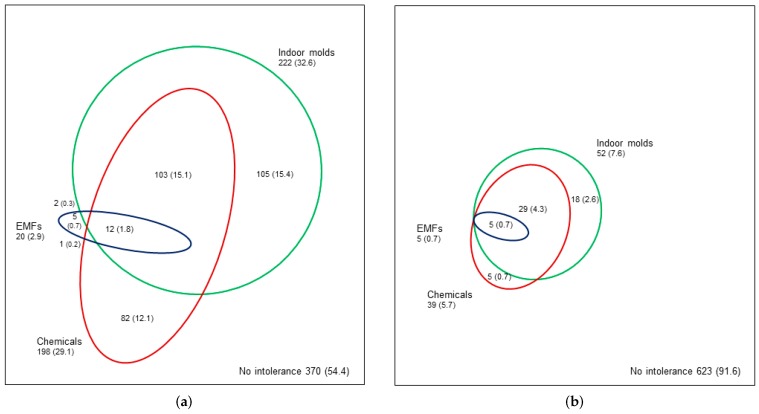
Co-occurrence of self-reported environmental intolerance (EI) attributed to chemicals, indoor molds, and electromagnetic fields (EMFs). In (**a**), EI definition A is used (annoyance attributed to two or more of the six chemicals in Table 1, indoor molds, or EMFs). In (**b**), EI definition E is used (definition A with multiple organ symptoms including central nervous system symptoms, behavioral changes, and disability). The percentage is calculated from the total *n* = 680, *n* (%).

Co-occurrence of the three EIs (chemicals, indoor molds, EMFs) is shown in Figure 2. Of all respondents, 17.4% reported co-occurrence of two or three types of EI definition A (annoyance), and 1.8% reported co-occurrence of all the three EIs (Figure 2a). Using EI definition E (annoyance with symptoms, behavioral changes, and disability), 5% reported co-occurrence of two or three types of EI, and 0.7% reported co-occurrence of all the three EIs (Figure 2b).

Of the respondents who reported EI definition A to chemicals (*n* = 198), indoor molds (*n* = 222), or EMFs (*n* = 20), 59%, 53%, and 75%, respectively, reported at least one other type of EI (Figure 2a). Furthermore, co-occurrence of at least one other EI, according to definition E, to chemicals, indoor molds, or EMFs, was reported by 87%, 65%, and 100%, respectively (Figure 2b). 

The association between the severity of disability and the co-occurrence (only one, two different types of EI, or all three EIs) of the three EIs (chemicals, indoor molds, EMFs) (*n* = 102, *p* = 0.037; Table 4) was statistically significant. This means that the more severe the disability, the more the EIs overlapped (Table 4). Increasing severity of the continuum of EI (definitions A–F) associated with the co-occurrence of the three EIs (*n* = 310, *p* < 0.001). 

## 4. Discussion

Our study illustrates the continuum of EI from annoyance to significant disability, which in numerous previous studies has appeared as varying prevalence rates depending on the definition used [14]. Among fertile-aged women, the prevalence rate of EI attributed to chemicals, indoor molds or EMFs varied from 46% (annoyance) to 0.9% (extreme difficulties at work, home, or in one’s social life). The prevalence of EI to chemicals, indoor molds, or EMFs, including at least some difficulties, was 5.7%, 7.6%, and 0.7%, respectively. The more difficulties were experienced, the more organ systems were involved, the more behavioral changes occurred, and the more the three EIs overlapped. All those who reported very much or extreme difficulties (2.2%) attributed EI to indoor molds, and two thirds to both molds and chemicals. 

EI can cause considerable individual suffering with socioeconomic implications, which has previously been shown in clinical populations (e.g., [28]). Berg et al. [1] studied behavioral changes due to chemical intolerance and reported that 3.3% of their participants had made adjustments to either their social lives or their occupational conditions, and 0.5% to both. In a general population cohort from the US, 1.5% reported losing their jobs and 0.8% reported moving house because of their hypersensitivity to chemicals [15]. These results are in line with our findings of 1.3% who had moved to another apartment and changed workplace due to various forms of EI, not only EI to chemicals. In our study, EI that causes difficulties in daily life was surprisingly prevalent and should be differentiated from annoyance, which was less disabling and encountered by half the population.

In earlier studies, disability due to EI has been described by, for example, symptoms and behavioral changes [1]. In our study, we used the tenth additional item of the PHQ-9, which was originally a single severity measure in the depression scale, to illustrate the severity of EI disability. This single item enabled us to identify individuals with severe difficulties, who are only a minority of those with EI. In a primary care sample this item has shown to correlate strongly with impairment in the domains of health-related quality of life [27]. The question measures functioning (activity and participation) in daily life. This question might also be a valuable method for clinical evaluation, as no laboratory test or other objective means is available for disability evaluation. In our study, an increase in the grade of disability (based on the single item from the PHQ-9) associated with the number of organ systems involved, the number of behavioral changes, and the co-occurrence of various types of EIs.

Few studies have assessed which environmental factors dominate in severe EI. Palmquist et al. [6] found that physician-diagnosed EI, representing a more severe EI, to chemicals was more common than EI to certain buildings, or to EMFs. In our study, the most severe end of the EI continuum was associated with indoor molds. These may reflect the general concern that indoor molds are an environmental health hazard in Finland. In a study of a working population in Finland, 11.4% perceived their workplaces’ indoor environments to be harmful from molds [29]. 

Our study further emphasizes that the prevalence of EI depends on which point on the severity continuum the definition of EI is focused. Almost all the respondents reported being at least somewhat annoyed by an environmental factor. The female gender (e.g., [1,16]), early pregnancy (e.g., [30]), and the wide range of enquired environmental factors may explain the high prevalence of annoyance. Different prevalence rates were found when EI included severe ratings of annoyance, symptoms, behavioral changes, or disability. These dimensions represent different features of intolerance, seen as a continuum with increasing severity. Our study succeeded in demonstrating this phenomenon in more detail than previous studies [1,6,15,16,19,20,22].

Similarity in the symptom spectrum of the three various forms of EI suggests that the different EIs represent the same phenomenon. This is also supported by the co-occurrence of EIs; if one EI was reported, another EI was reported by more than half of the respondents. An overlap of various EIs has also been shown previously [6,16]. Our study showed that more co-occurrence was seen with increasing severity of EI. We found multiple organ symptoms to be prevalent in EI. Multiple organ symptoms, including CNS symptoms, are characteristic of EI and all other functional disorders [4,31]. 

The high number of symptoms is associated with functional impairments in individuals with EI [32], reflecting the severity of the condition. These findings strengthen the understanding that EI is similar to functional disorders (e.g., fibromyalgia, chronic fatigue syndrome), which share common mechanisms, i.e., sensitization of CNS due to dysfunctional cognitions [33]. When individuals perceive certain environmental factors as health hazards, stress reactions manifest as multiple organ symptoms [8]. The perceptions of the risks associated with environmental pollutants may contribute to the development of EI [34]. 

Our study focused on all fertile-aged women attending a birth clinic of the Kuopio central hospital region. We focused on women because they typically report EI more often than men [2,13]. Our study represents an age group in which EI is prevalent [2]. Increased perception of odors and unpleasant qualities is encountered particularly in early pregnancy [30], which may have increased the reporting of EI in our study. To avoid excess reporting, the respondents were asked to evaluate the time prior to their pregnancy, not limiting to a certain period of time. These may be sources of information bias. If early pregnancy increases the reporting of annoyance, it is not plausible that it could increase the number of individuals reporting severe difficulties due to EI. Another potential source of bias is the paucity of background information. The findings of previous studies concerning the association between education and EI are inconsistent [2,13]. Concomitant diseases were not the focus of this study, but it is known that several different diseases (e.g., asthma, mental disorders) and functional disorders associate with EI, although none of them entirely explains it. Regardless of concomitant somatic or mental diseases, the important factor is whether individuals attribute their symptoms, behavioral changes and disability to the environment.

The low proportion of respondents can be considered a limitation of the study. We were able to recruit 27% of the pregnant women of the maternity clinic clients in the region. The results may exaggerate the prevalence rates, as individuals with environment-related annoyance are more likely to participate in a study investigating environmental issues. Thus, although the results represent EI among fertile-aged women, the low participation rate calls for caution in the generalization of the results.

Recent data on the natural course of EI show reversibility of EI [35]. During a six-year period, one fifth of the individuals with EI recovered, especially those with less affective and behavioral changes [35]. So far, EI has been considered a chronic condition and resistant to therapy [2,4,36], but there is a lack of research on the course of EI and on interventions aimed at reducing reactivity to the environment. Similarities to and shared mechanisms with functional disorders offer opportunities for prevention and recovery even in severe EI [8].

## 5. Conclusions

EI is a continuum from annoyance, which is frequent, to severe disability. Our findings support previous studies suggesting that EI shares features with functional disorders. Interventions proven efficient for functional disorders may be useful in EI with disability. Thus, better recognition is needed.

## Figures and Tables

**Table 1:** Questions to assess environmental intolerance.

Item	Question and Answer Options
Annoyance	Are you feeling ill or annoyed by the following types of environmental exposures or situations?
Chemicals (1)–(6)	(1) Vehicle exhaust (2) Paint or paint thinner (3) Perfumes, air fresheners or other fragrances (4) New furnishings such as new carpeting, flooring, shower curtain, or the interior of a new car (5) Fresh ink on newspapers (6) Tobacco smoke
Indoor molds (7)	(7) Indoor molds in moisture-damaged buildings
EMFs (8)	(8) Electromagnetic fields
Other environmental factors (9)–(12)	(9) Beauty salons or hair salons (10) Detergent departments in shops (11) Moldy odors (12) Dust
Sensitivity	Are you exceptionally/unusually sensitive to the environmental exposures or situations above?
Symptoms	Have you ever had the following symptoms from the environmental exposures or situations listed above? - Neurological symptoms (e.g., headache, numbness, tingling) - Cognitive symptoms (e.g., memory deterioration, concentration impaired) - Pulmonary symptoms (e.g., dyspnea, coughing, wheezing) - Dermal symptoms (e.g., erythema, rash) - Muscles or joint pain - Gastrointestinal symptoms (e.g., flatulence, stomach ache) - Cardiac symptoms (e.g., palpitations) - General symptoms (e.g., fever, night sweats, fatigue, weight loss, increase in weight)
Behavioral changes	Have you made any behavioral changes to avoid the symptoms above? - Behavior or lifestyle change to minimize exposure - Changed interior decorations or furnishings at home - Moved to another apartment - Changed workplace, resigned from workplace or occupation - Taken vitamins, nutritional supplements, or changed diet - Eliminated the cause using antifungal agents, or chemicals - Used protective equipment (e.g., respirator, gauntlet, clothing)
Disability	If you recognize the problems mentioned above, how difficult have these problems made it for you to do your work, take care of things at home or get along with other people?

Items 1–6 are based on Black et al. [26]; items 9–10 are based on Kreutzer et al. [20].

**Table 2:** Prevalence of degree of annoyance attributed to various environmental factors (total *n* = 680), *n* (%).

Environmental Factor	Degree of Annoyance *			
	Not at All	Some	Rather Much	Very Much
Chemicals				
Vehicle exhaust	263 (38.7)	324 (47.7)	71 (10.5)	21 (3.1)
Paint or paint thinner	211 (31.4)	324 (48.1)	110 (16.3)	28 (4.2)
Perfumes, air fresheners, or other fragrance	273 (40.3)	258 (38.0)	117 (17.3)	30 (4.4)
New furnishings such as new carpeting, flooring, shower curtain, or the interior of a new car	441 (65.7)	190 (28.3)	33 (4.9)	7 (1.1)
Fresh ink of newspapers	544 (80.0)	110 (16.2)	23 (3.4)	3 (0.4)
Tobacco smoke	133 (19.6)	211 (31.1)	175 (25.8)	159 (23.5)
Indoor molds	200 (29.7)	252 (37.4)	148 (21.9)	74 (11.0)
Electromagnetic fields	568 (84.7)	83 (12.4)	15 (2.2)	5 (0.7)
Other factors				
Beauty salons or hair salons	468 (68.8)	175 (25.8)	28 (4.1)	9 (1.3)
Shop detergent departments	487 (71.7)	146 (21.5)	39 (5.8)	7 (1.0)
Moldy odors	217 (32.1)	300 (44.3)	114 (16.8)	46 (6.8)
Dust	178 (26.4)	310 (45.9)	147 (21.8)	40 (5.9)

* Missing values (*n* = 0–9 per item) have been excluded.

**Table 3:** Prevalence of environmental intolerances (EIs) according to EI definitions A–F * used in this study. An individual may have EI to one or more factors and may be included in various definitions A–F. The percentage is calculated from the total *n* = 680, *n* (%).

Definitions of EI	EI Attributed to				
	Any of the 12 Environmental Factors	Chemicals **, Molds, or EMFs (Any of the Three)	Chemicals **	Molds	EMFs
A	457 (67.2)	310 (45.6)	198 (29.1)	222 (32.6)	20 (2.9)
B	302 (44.4)	226 (33.2)	155 (22.8)	166 (24.4)	16 (2.4)
C	145 (21.3)	119 (17.5)	80 (11.8)	93 (13.7)	9 (1.3)
D	122 (17.9)	102 (15.0)	67 (9.9)	83 (12.2)	9 (1.3)
E	68 (10.0)	57 (8.4)	39 (5.7)	52 (7.6)	5 (0.7)
F	15 (2.2)	15 (2.2)	10 (1.5)	15 (2.2)	2 (0.3)

EMFs, Electromagnetic fields; * Definitions are described in the Methods section; ** Two or more of the six chemicals in Table 1.

**Table 4:** Distribution of the degree of disability in those with one, two, or three types of environmental intolerance (EI attributed to chemicals *, indoor molds, or EMFs), *n* (%) **.

Number and Type of EI	Degree of Disability (Lifestyle or Functional Impairments) ***				
	Not Difficult at All	Somewhat Difficult	Very Difficult	Extremely Difficult	Total
Only one EI	30 (56.6)	18 (33.9)	3 (5.7)	2 (3.8)	53 (100)
Chemicals only	13 (72.2)	5 (27.8)	-	-	18 (100)
Indoor molds only	16 (47.1)	13 (38.2)	3 (8.8)	2 (5.9)	34 (100)
EMFs only	1 (100)	-	-	-	1 (100)
Two different types of EI	12 (29.3)	21 (51.2)	5 (12.2)	3 (7.3)	41 (100)
Chemicals and molds	12 (29.3)	21 (51.2)	5 (12.2)	3 (7.3)	41 (100)
Chemicals and EMFs	-	-	-	-	-
Molds and EMFs	-	-	-	-	-
All three (chemicals, molds, EMFs) EIs	3 (37.5)	3 (37.5)	1 (12.5)	1 (12.5)	8 (100)

EMFs, Electromagnetic fields; * Two or more of the six chemicals in Table 1; ** Only cases who reported EI attributed to chemicals, molds, or EMFs with multiple organ symptoms including central nervous system symptoms, and any behavioral changes (fulfill at least EI definition D) are included (*n* = 102); *** The single item is from the PHQ-9 questionnaire [27].
